# Supplementary material for: Evaluation of new strain (AAD16) of Beauveria bassiana recovered from Japanese rhinoceros beetle: Effects on three coleopteran insects
Source: PLoS One. 2024 Jan 10;19(1):e0296094. doi: 10.1371/journal.pone.0296094 (PMC10781193; doi:10.1371/journal.pone.0296094)
Supplement: Attachment — Submitted filename: Evaluation of new strain (AAD16) of Beauveria bassiana.docx [file pone.0296094.s002.docx]

*Manuscript Title:* **Evaluation of new strain (AAD16) of** Evaluation of new strain (AAD16) of Beauveria bassiana recovered from Japanese rhinoceros beetle: effects on three coleopteran insects**recovered from Japanese rhinoceros beetle: effects on three coleopteran insects**

General Comments: (**Minor Comments)**

The manuscript titled " **Evaluation of new strain (AAD16) of Beauveria bassiana recovered from Japanese rhinoceros beetle: effects on three coleopteran insects** " explores the potential of new *Beauveria* species as biological control agents against the three **coleopteran insects**. The study addresses a relevant research area and offers important insights for pest management strategies using biological averages. This study confirms a broad spectrum of *Bauveria* species as entomopathogenic fungi.

The manuscript is generally well structured, and the experiments are carefully designed. However, a few points require improvement before the manuscript can be considered suitable for publication in PLOS ONE.

- Line 4: replace “….its virulency” with “its virulence”,
- Line 5: change “on three coleopteran..” by “focusing on its effect on three coleopteran”,
- Line 7: 15.3 !! After comma, two number is usually used to convey precision without overwhelming the reader. try to apply this in all documents!
- Line 7 : replace “ and 19,4% lower on strain AAD16 » by and 19,4% lower for strain AAD16
- Line 9 : replace “1.3 and 1.2 times higher than strain ARP14” by “1.3 and 1.2 times higher than that of strain ARP14”
- Line 11 : replace “47.1% lower on strain “ by “47.1% lower for strain”
- Line 13 : replace “1.8 higher than strain” by “1.8 higher than that of strain”
- Line 14 : replace ‘’ the strain AAD16 also showed higher mortality (90.0%) for larvae of A. dichotoma than did the strain ARP14’’ by ‘’the strain AAD16 also showed higher larval mortality (90%) for A. dichotoma compared to strain ARP14’’
- Line 25 : The Latin names of the species must be written entirely the first time in the text (ex: *Beauveria bassiana* (line 25). After the following repetitions (line 30, 48 ….) they can be abbreviated on *B. bassiana*, and this applies equally to all other species mentioned in the text.
- Line 60 : added the company of the Sabouroud Dextrose Agar
- Line 61 : added the conditions of incubation ( T° , RH , photoperiod)
- Line 64 : We isolated a single colony of the fungus and transferred after 72 h… into what  (medium ) ??
- Line 69 : replace “53± 0.9%”	by “53 ± 0.9 % RH “
- Line 66 : replace “Insect Source” by “Insect source”
- Line 76 : replace “and 24.9±0.00°C and 52.3± 0.8% RH for with a photoperiod of 16 :8h (L :D)’’ by “and 24.9±0.00°C and 52.3± 0.8% RH for *A. dichtoma* with a photoperiod of 16 :8h (L :D)
- line 79 : replace ‘’ Morphological Identification of B. bassiana strains ‘’ by Morphological “identification of B. bassiana strains”
- line 115 : in this paragraph its better to mention the formula used to calculate the mortality of larva
- line 119 : replace “petri dishes” by “Petri dishes”
- line 119 : did you used the sterilized Petri dishes or non-sterilized ?
- line 120 : homogenize the writing of photoperiod conditions in the all of document, 16 :8( L : D)
- line 124 : in this paragraph, it's better to mention the formula used to calculate the larva and adult mortality of M. alternatus
- line 127 : replace “were tested” by “were used”
- line 129 : replace “petri” by “Petri”
- line 166 : replace *Beauveria bassiana* by *B . bassiana*
- line 174 : replace “at 6,8 and 19 days after exposure” by “at 6,8 and 19 days, respectively, after exposure”
- Figures and tables are generally well-presented and support the findings. However, it is advisable to include more descriptive captions (statistic information (P-value)) that succinctly explain the content
- Figure legends 3-6 : the absence of standard error bars of the mean and asterisk's meaning.
- Reference: line 245: Citation number 45 is missing in the text. Please ensure that all references are cited according to the required journal style.

The manuscript could provide valuable information in the field of pest management. However, major revisions are needed (minor comments mentioned above). It is recommended that the authors revise the manuscript accordingly and provide additional details in the methodology section to improve the rigor of the study and its reproducibility for future researchers. Thank you for the opportunity to review this work. I look forward to seeing the revised manuscript and the contributions it will make to the field.

Sincerely,
