# Supplementary material for: Evaluation of new strain (AAD16) of Beauveria bassiana recovered from Japanese rhinoceros beetle: Effects on three coleopteran insects
Source: PLoS One. 2024 Jan 10;19(1):e0296094. doi: 10.1371/journal.pone.0296094 (PMC10781193; doi:10.1371/journal.pone.0296094)
Supplement: Attachment — Submitted filename: Point-by-point response to reviewers (1st revision).docx [file pone.0296094.s003.docx]

***** 1. Response to editor’s comments**

1. Mycosis observation on died insects

>> Yes. We observed the mycosis development on dead insects. We addressed this issue in our previous MS in figure 4 and 6.

2. Was the fungal strain re-isolated from the insect that undergone mycosis?

>> Yes, for confirmation purposes, we only re-isolated the fungus from *T*. *molitor* and checked for molecular identification using the ITS primer which confirmed our targeted organism.

3. On which larval stages of insects, was the bioassay done?

>> It was very difficult to separate the larval stages due to long larval period, thus overlapped larval stages. Thus, we provided the larval sizes for *T*. *molitor* and *A*. *dichotoma* and the larval age for *M*. *alternatus* (see L121, L132, and L144 in the revised MS).

***** 2. Response to reviewer #1’s comments**

>> We found mismatch in line numbers reviewer indicated, thus we provided the line numbers of the first MS in parenthesis with red font next to the line numbers reviewer indicated.

1. The manuscript titled " Evaluation of new strain (AAD16) of Beauveria bassiana recovered from Japanese rhinoceros beetle: effects on three coleopteran insects " explores the potential of new Beauveria species as biological control agents against the three coleopteran insects. The study addresses a relevant research area and offers important insights for pest management strategies using biological averages. This study confirms a broad spectrum of Beauveria species as entomopathogenic fungi.

>> Thanks for the comments.

2. Line 4: replace “….its virulency” with “its virulence”,

>> Replaced “its virulency” to “its virulence” (see L04 in the revised MS).

3. Line 5: change “on three coleopteran..” by “focusing on its effect on three coleopteran”,

>> Replaced “on three coleopteran” to “focusing on its effect on three coleopteran” (see L05-06 in the revised MS).

4. Line 7: 15.3 !! After comma, two number is usually used to convey precision without overwhelming the reader. try to apply this in all documents!

>> We set three significant figures for the data.

5. Line 7: replace “ and 19,4% lower on strain AAD16 ? by and 19,4% lower for strain AAD16

>> Replaced “and 19.4% lower on strain AAD16” to “and 19.4% lower for strain AAD16” (see L07-08 in the revised MS).

6. Line 9: replace “1.3 and 1.2 times higher than strain ARP14” by “1.3 and 1.2 times higher than that of strain ARP14”

>> Replaced “1.3 and 1.2 times higher than strain ARP14” to “1.3 and 1.2 times higher than that of strain ARP14” (see L09-10 in the revised MS).

7. Line 11 : replace “47.1% lower on strain “ by “47.1% lower for strain”

>> Replaced “47.1% lower on strain” to “47.1% lower for strain” (see L12 in the revised MS).

8. Line 13 : replace “1.8 higher than strain” by “1.8 higher than that of strain”

>> Replaced “1.8 higher than strain” to “1.8 higher than that of strain” (see L13 in the revised MS).

9. Line 14: replace ‘’ the strain AAD16 also showed higher mortality (90.0%) for larvae of A. dichotoma than did the strain ARP14’’ by ‘’the strain AAD16 also showed higher larval mortality (90%) for A. dichotoma compared to strain ARP14’’

>> Replaced “The strain AAD16 also showed higher mortality (90.0%) for larvae of *A*. *dichotoma* than did the strain ARP14” to “The strain AAD16 also showed higher larval mortality (90%) for *A*. *dichotoma* compared to strain ARP14’” (see L14-15 in the revised MS).

10. Line 25: The Latin names of the species must be written entirely the first time in the text (ex: Beauveria bassiana (line 25). After the following repetitions (line 30, 48 ….) they can be abbreviated on B. bassiana, and this applies equally to all other species mentioned in the text.

>> Replaced “*Beauveria bassiana*” to “*B*. *bassiana*” (see L30, 106, and 176 in the revised MS).

11. Line 60: added the company of the Sabouroud Dextrose Agar

>> Added the company name in the revised MS (see L63 in the revised MS).

12. Line 61: added the conditions of incubation ( T° , RH , photoperiod)

>> We addressed this issue in our previous MS in L103-104 (see L106-107 in the revised MS).

13. Line 64: We isolated a single colony of the fungus and transferred after 72 h… into what (medium ) ??

>> Added “in SDA media” (see L67 in the revised MS).

14. Line 69: replace “53± 0.9%” by “53 ± 0.9 % RH “

>> Replaced “53± 0.9%” to “53.0 ± 0.9% RH” (see L72 in the revised MS).

15. Line 66: replace “Insect Source” by “Insect source”

>> Replaced “Insect Source” to “Insect source” (see L69 in the revised MS).

16. Line 76: replace “and 24.9±0.00°C and 52.3± 0.8% RH for with a photoperiod of 16 :8h (L :D)’’ by “and 24.9±0.00°C and 52.3± 0.8% RH for A. dichtoma with a photoperiod of 16 :8h (L :D)

>> Replaced “and 24.9±0.0°C and 52.3± 0.8% RH for with a photoperiod of 16 :8h (L :D)” to “and 24.9 ± 0.0°C and 52.3 ± 0.8% RH for *A*. *dichotoma* with a photoperiod of 16:8 hour (Light:Dark)” (see L79-80 in the revised MS).

17. line 79: replace ‘’ Morphological Identification of B. bassiana strains ‘’ by Morphological “identification of B. bassiana strains”

>> Replaced “Morphological Identification of *B*. *bassiana* strains” to “Morphological identification of *B*. *bassian*a strains” (see L82 in the revised MS).

18. line 115: in this paragraph its better to mention the formula used to calculate the mortality of larva

>> We believe it is OK as we provided reference (see L152 in the revised MS).

19. line 119 (L120): replace “petri dishes” by “Petri dishes”

>> Replaced “petri dishes” to “Petri dishes” (see L123 in the revised MS).

20. line 119 (L120): did you used the sterilized Petri dishes or non-sterilized ?

>> We did not sterilize the Petri dishes by ourselves, we just used brand-new (pre-sterilized) Petri dishes in all bioassays.

21. line 120 (L121): homogenize the writing of photoperiod conditions in the all of document, 16 :8( L : D)

>> Changed “16 L: 8 D” to “16:8 hour (Light:Dark)” (see L72, L124-125, 136, and L147 in the revised MS).

22. line 124: in this paragraph, it's better to mention the formula used to calculate the larva and adult mortality of M. alternatus

>> We believe it is OK as we provided reference (see L152 in the revised MS).

23. line 127 (L128): replace “were tested” by “were used”

>> Replaced “were tested” to “were used” (see L133 in the revised MS).

24. line 129 (L130): replace “petri” by “Petri”

>> Replaced “petri” to “Petri” (see L135 in the revised MS).

25. line 166 (L167): replace Beauveria bassiana by B . bassiana

>> Replaced “*Beauveria bassiana*” to “*B*. *bassiana*” (see L176 in the revised MS).

26. line 174: replace “at 6,8 and 19 days after exposure” by “at 6,8 and 19 days, respectively, after exposure”

>> Replaced “at 6, 8 and 19 days after exposure” to “at 6, 8, and 19 days, respectively, after exposure” (see L183-184 in the revised MS).

27. Figures and tables are generally well-presented and support the findings. However, it is advisable to include more descriptive captions (statistic information (P-value)) that succinctly explain the content

>> Statistic information with P-values were added in figure captions (see L440-441, L443-445, L447-448, L450-451, and L453 in the revised MS).

28. Figure legends 3-6: the absence of standard error bars of the mean and asterisk's meaning.

>> We did the Chi-square test using the total number of insects; that’s why there are no standard errors. We explain the meaning of the asterisk sign in the figure caption (see L440-441, L443-445, L447-448, and L450-451 in the revised MS).

29. Reference: line 245: Citation number 45 is missing in the text. Please ensure that all references are cited according to the required journal style.

>> The citation number 45 was presented in the first MS in L232 (see L241 in the revised MS).

***** 3. Response to reviewer #2’s comments**

1. This research work presented minimum amount of data and lacks of novelty. Only one Beauveria bassiana isolate was isolated and characterized and compared its efficacy with an in-house isolate against larvae of three insect through a single bioassay test. For molecular characterization, ITS region was used, but using more specific region like TEF would be good for this type pleomorphic fungus. However, my main concern is that the method of fungal inoculation by using a syringe is inappropriate and practically impossible in field use. The virulence of entomopathogenic fungi includes the invasion capabilities through insect cuticle through germination on host body. This manuscript did not show any visual evidence of mycosed larvae which is essential. In addition, the following question need to be addressed.

>> We agree with the reviewer’s comment. Firstly, we compared the virulence of our new fungus strain (AAD16) with another *B*. *bassiana* strain (ARP14) because, in our previous study, we found *B*. *bassiana* ARP14 is more virulent than the commercial strain *B*. *bassiana* GHA on hemipteran and lepidopteran insects. We mentioned this issue in the first MS in L49-51.

Secondly, yes, using a syringe is inappropriate and practically impossible in field use. As our intention was to compare the efficacy of two fungus strains, we wanted to apply the same amount of conidia from both fungi to the insect body using a microsyringe.

2. Was the fungal strain re-isolated from the insect that undergone mycosis?

>> Yes, for confirmation purposes, we only re-isolated the fungus from *T*. *molitor* and checked for molecular identification using the ITS primer which confirmed our targeted organism.

3. On which larval stages of insects, was the bioassay done?

>> It was very difficult to separate the larval stages due to long larval period, thus overlapped larval stages. Thus, we provided the larval sizes for *T*. *molitor* and *A*. *dichotoma* and the larval age for *M*. *alternatus* (see L121, L132, and L144 in the revised MS).

4. What is the effect of this strain on other larval stages? Is there any sub-lethal effect?

>> We did not test larvae other than described in the text nor evaluate the sublethal effect.

***** 4. Response to reviewer #3’s comments**

>> We found mismatch in line numbers reviewer indicated, thus we provided the line numbers of the first MS in parenthesis with red font next to the line numbers reviewer indicated.

1. Rewrite the objective of your study

>> We rewrite the objective as " In this study, we report a new strain of *B*. *bassiana*, designated AAD16, and then we determine the relative pathogenicity of the new isolate (AAD16) against three coleopteran insects, i.e., *Tenebrio molitor* L. (Coleoptera: Tenibrionidae), *Monochamus alternatus* Hope (Coleoptera: Cerambycidae), and *Allomyrina dichotoma* (L.) (Coleoptera: Scarabaeidae), compared to isolate *B*. *bassiana* ARP14 in a laboratory bioassay. *B*. *bassiana* ARP14 is effective against different hemipteran and lepidopteran insects [17-19]” (see L43-48 in the revised MS).

2. Line 42 from “in this study” to line 50 “insects” should be in Materials and methods section

>> Deleted L43-51 from the previous MS and made a new section in Materials and Methods in the revised MS (see L54-59 in the revised MS). We also deleted L58-59, “An adult *A*. *dichotoma* infected with *B*. *bassiana* was collected from field in Andong, Republic of Korea in 2016 (36.550909, 128.802944),” in the first submission.

3. In each experiment, how much larvae and/or adults were used

>> Added larval or adult number (see L125-127, L137-139, and L148 in the revised MS).

4. Indicate how much repetition in each experiment

>> Added number of replication (see L125-127, L137-139, and L148 in the revised MS).

5. Homogenate 16: 8 (L:D)

>> Changed “16 L: 8 D” to “16:8 h (Light:Dark)” (see L72, L124-125, 136, and L147 in the revised MS).

6. The h, d, D, L, dia and RH should be clear.

>> h, d, D, L, dia and RH changed to hour, day, Dark, Light, diameter, and relative humidity, respectively ( see L9, 13, 15, 64, 67, 72, 80, 85, 107, 124, 128, 135, 136, 139, 146, 147, 149, 179, 180, 185, 189, 191, 194, 198, 200, 202, 203, 205, 207, 212, 214, 216, 233, 244, 248, 252, Table 2-4, 446, and 449 in the revised MS).

7. The English need a revision by a mother tongue to improve the language.

>> We made the first MS revised by a commercial company ([www.vandrieschescientificediting.com](http://www.vandrieschescientificediting.com)) as we indicated in the first submission. If this revised MS still needs English revision, we will submit it to the company again. Please let us know.

8. L22: change including fungus…by including fungi

>> Changed “fungus” to “fungi” (see L22 in the revised MS).

9. L32: but also on other parameters

>> We couldn’t understand this comment. Please rephrase it in next revision.

10. L56: Isolation and mass production of the pathogen Beauveria bassiana

>> Changed “Isolation and mass production of the pathogen” to “Isolation and mass production of the pathogen *B*. *bassiana*” (see L61 in the revised MS).

11. L69: 0.9% RH

>> Added “RH (relative humidity)” (see L72 in the revise MS).

12. L75: delete provided by the company

>> Deleted “provided by the company” (see L78 in the revised MS).

13. L 92: under the conditions followed: under the following conditions

>> Changed “under the following conditions” to “under the conditions followed” (see L95-96 in the revised MS).

14. L119: (L120): Petri dishes

>> Changed “petri dishes” to “Petri dishes” (see L123 in the revised MS).

15. L131 and L 138 (L139): Triton X-100 ddH2O (0.1%) used as a control

>> We corrected as “Triton X-100 ddH2O (0.1%) was used as a control” (see L136-137 and L147-148 in the revised MS).

16. L194 (L195): change “ for the” by “ the ones obtained with”

>> Changed “for the” to “the ones obtained with” (see L204-205 in the revised MS).

17. L225 (L226): we found that the B. bassiana AAD16 strain killed

>> We deleted “we found that” (see L236 in the revised MS).

18. L227 B. bassiana ARP14, caused only only 69% mortality

>> Deleted “only” (see L238 in the revised MS).

*** 5. Authors’ self-corrections (Line numbers are from the first MS)

L62: Deleted space (see L65 in the revised MS)

L69: Added “(degree Celsius)” (see L72 in the revised MS).

L93,108: Changed “min” to “minute” (see L96 and L111 in the revised MS).

L121, 130: Changed “h” to “height” (see L124 and L135 in the revised MS).

L178, 182: Deleted space (see L187 and L191 in the revised MS).

L184: Added space (see L193 in the revised MS).

L258: Deleted space (see L268 in the revised MS).

L275-277: We added “Supporting information”

Table 2: Deleted “(n= 20)” from the caption and added a column for “n”

Figure 3, 7: Changed “d” to “days” (see Figure 3 and Figure 7 in the revised MS).

Figure 5, 6: Changed “h” to “hours” (see Figure 5 and Figure 6 in the revised MS).
